# Hospitalization Rates and Discharge Status in Multiple Sclerosis

**DOI:** 10.1155/2013/436929

**Published:** 2013-05-13

**Authors:** Sanober Nusrat, David Levinthal, Klaus Bielefeldt

**Affiliations:** University of Pittsburgh Medical Center, Division of Gastroenterology, 200 Lothrop Dr., Pittsburgh, PA 15213, USA

## Abstract

Management of multiple sclerosis (MS) has shifted from supportive to disease modifying therapy. Considering the increasingly widespread adoption of this approach in managing MS patients, we hypothesized that hospitalizations and surrogates of disease-related complications should have declined during the last decade. *Methods*. Using the Nationwide Inpatient Sample, hospitalizations for MS and associated secondary diagnoses and procedures as well as discharge status were examined. Time trends were examined for different age cohorts focusing on the period from 2001 to 2010. *Results*. During the preceding decade, annual hospitalizations for MS increased by 40%, with stable rates in all age groups except geriatric patients, who accounted for a significantly higher fraction of admissions. Nursing home transfers as a surrogate marker of disability remained unchanged for all age groups. Similarly, urinary tract infections, the need for skin debridement, or gastrostomy tube placement did not vary during the decade. *Conclusion*. During a time of increased adoption of disease modifying therapy, MS-related hospitalizations continued to increase and surrogate measures of disability in admitted patients remained stable, demonstrating the still significant impact of the disease on affected individuals.

## 1. Introduction

Multiple sclerosis (MS) tends to have a progressive course with gradual loss of function in many affected individuals. Within about 10 years, 25–30% of the affected individuals will require a cane or wheelchair to compensate restricted mobility [1–3]. With loss of lower extremity strength, many patients also develop anorectal, urinary, and sexual dysfunction [4–6]. We have recently reviewed the prevalence of anorectal dysfunction in MS [7]. While different recruitment strategies and definitions of key endpoints complicate the comparison of published results, constipation and fecal incontinence are commonly reported with rates being relatively stable over time. These results appear to be consistent with studies on the prevalence of urinary symptoms and problems due to neurogenic bladder. Urgency or urinary incontinence remain common affecting 10–50% of the patients and increasing their risk of urinary tract infection and hospitalization [5, 6, 8–10].

The consistently high likelihood of experiencing problems due to neurogenic bladder or bowel dysfunction stands in apparent contrast with the presumed benefits of disease modifying therapy (DMT) that has been introduced about one decade ago. Initial studies of glatiramer acetate described a 30% reduction of relapse rates that was maintained over time [11–13]. Similarly, therapy with interferon *β*1 reduced flares and delayed progression [14, 15]. These results led to the approval of these agents for the management of MS with the hope to limit the long-term impact with increasing disability [16]. Several other orally or parenterally administered agents have been tested with similar effects on newly occurring brain lesions and/or clinical disease progression [17–20]. Since its first introduction, the use of such disease modifying therapies has increased significantly, accounting for more than 60% of medication costs for MS [2, 21–25]. Considering the more widespread use of these therapies and the persistently high rates of symptoms due to neurogenic bladder or bowel problems, we decided to study time trends in hospitalization rates and discharge patterns due to MS, using the National Inpatient Sample of the Agency for Healthcare Research and Quality. The underlying hypothesis was that the increasing use of disease modifying therapy should decrease surrogate markers of disability. We specifically addressed the following questions. (1) Did the fraction of nursing home transfers decrease over time? (2) Did the age distribution of admissions and that of patients discharged to a nursing home change over time? (3) Did the rate of urinary tract infections as a potential complication of neurogenic bladder change over time?

## 2. Method

The Nationwide Inpatient Sample (NIS) of the Agency for Healthcare Research and Quality was searched for the decade spanning 2001 to 2010. The NIS contains a compilation of data from more than 1000 hospitals across the United States. Annual admissions were extracted using International Classification of Diseases (ICD) 9 code 340 for MS as primary or secondary diagnosis. Aggregate data were abstracted using the publically available datasets. Information about age cohorts, gender, discharge status, and associated diagnosis of urinary tract infection was abstracted. In addition, the fraction of admissions with associated procedures for cutaneous debridement or gastrostomy tube placement was retrieved; these procedures functioned as surrogate markers of decubital ulcerations and impaired oral intake, likely due to oropharyngeal dysphagia, respectively. Secondary diagnoses and associated procedures were bundled based on the Clinical Classification System of the Agency for Healthcare Research and Quality. All data are given as mean ± SEM. Group means were compared using ANOVA (Sigmastat 2.0; SPSS, Chicago). 

## 3. Results

**Figure 1:**
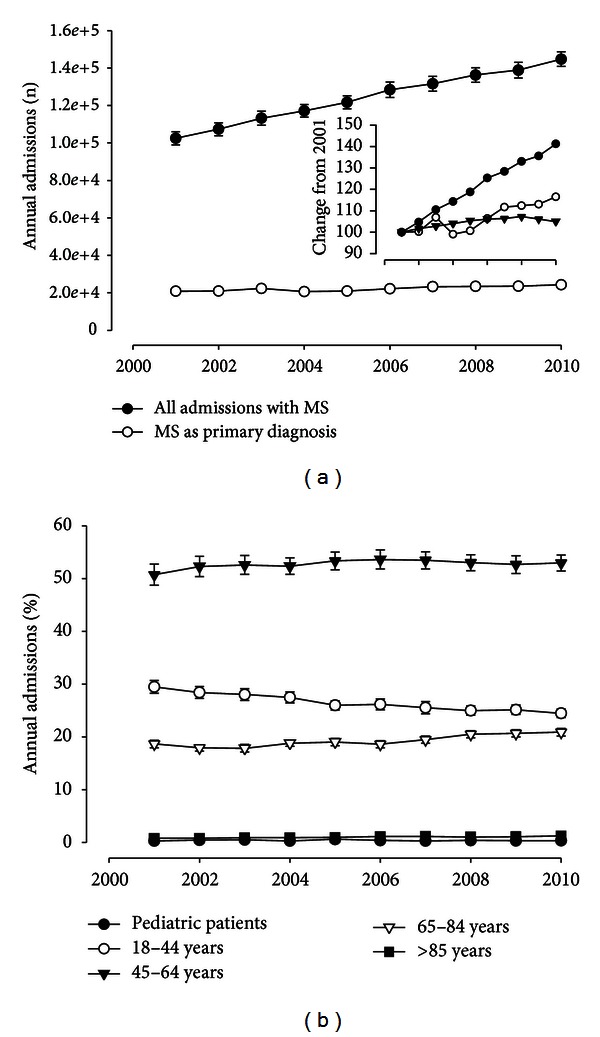
(a) Annual admissions for MS for the decade between 2001 and 2010 separated for MS as primary diagnosis (white circles) of all admissions that included MS as a diagnosis (black circles); the insert shows percentage of change from baseline in 2001 in comparison to all hospitalizations (triangles). (b) Relative age distribution of MS-related admissions during the 10-year period based on predefined age cohorts.

Within the decade of this study, annual admissions with MS as primary or secondary diagnosis increased from 102, 473 ± 3, 485 in 2001 to 144, 716 ± 3, 902 in 2010 (Figure 1(a)). This more than 40% increase compares to a 4% rise in overall hospitalizations within the same time frame (*P* < 0.001). Consistent with the known epidemiology of MS, women accounted for 73.1 ± 0.1% of the admissions. As changes in MS prevalence could potentially confound these findings [26], we next focused on the age distribution of hospitalized patients. The age cohort between 45 and 64 years accounted for more than 50% of the admissions, while there was a slight shift with fewer young adults (age cohort 18–44 years) and more old adults being admitted (Figure 1(b)), the relative decrease in younger adults did not reach significance (*P* = 0.87), while the rise in admissions of patients over 65 years of age was significant (*P* < 0.05). 

**Figure 2:**
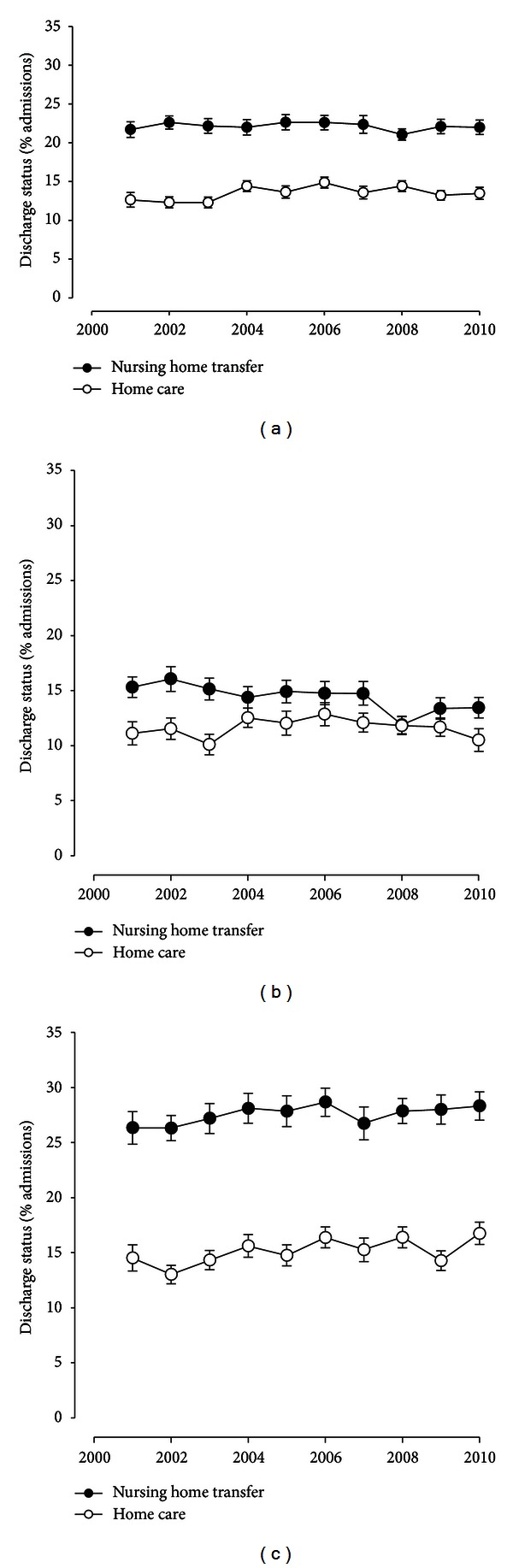
Time-dependent changes in discharge status as surrogate measures of functional impairment: nursing home transfers and discharge to ongoing home care are plotted for all the admissions with MS (a), admissions of patients 18–44 years (b) and 45–64 years (c).

Looking at nursing home transfers and the need for ongoing home care as surrogate markers of ongoing functional impairment, we next analyzed the discharge pattern over time. As shown in Figure 2, nursing home transfers (*P* = 0.54) and referrals to home care (*P* = 0.12) remained stable for the entire cohort and were higher in admissions of older patients (*P* < 0.001; Figure 2(c)). However, even admissions of younger adults resulted in nursing home transfers or home care referrals in about 15% and 12%, respectively, without significant changes during the time period examined (Figure 2(b); *P* = 0.88 and *P* = 0.79, resp.).

**Figure 3:**
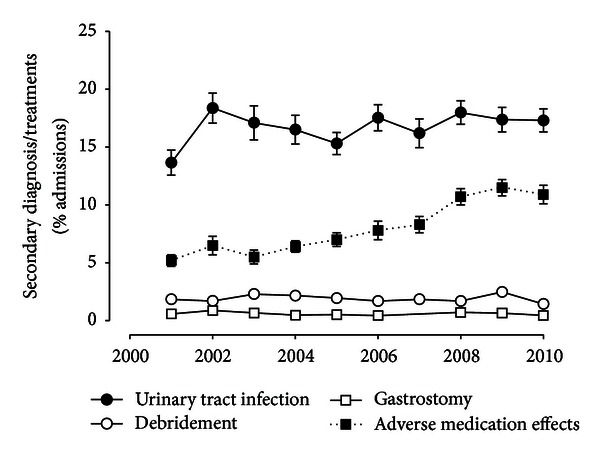
Time trends in surrogate measures of disease-related complications. The relative frequency of urinary tract infections (black circles) and adverse medication effects (black squares) as secondary diagnoses or wound debridement (white circles) and gastrostomy placement (white squares) were normalized for the total number of admissions and plotted as a function of time.

Considering stable rates of discharges into environments with ongoing medical and nursing care, we examined additional surrogate markers of functional impairment. Neurogenic bladder significantly increases the risk of urinary tract infections and hospitalizations [5, 8]. Using a secondary diagnosis of urinary tract infection as potential marker for neurogenic bladder, slightly less than 20% of the MS-related hospitalizations were associated with such an infection, with a significantly lower rate being reported only in 2001 (*P* < 0.01); in the subsequent years, the rate did not change over time (*P* = 0.39; Figure 3). Significant decubital ulcers have been observed in about 9% of MS patients admitted to nursing homes [27]. Using the need for debridement as an indicator for such cutaneous complications of advanced MS, about 2% of the admissions were associated with such interventions with stable rates during the decade studied (*P* = 0.73; Figure 3). Severe oropharyngeal dysphagia develops with progression of disability in MS [28, 29] and may necessitate enteral alimentation with gastrostomy tubes [30]. While less than 1% of the admissions were associated with gastrostomy tube placement, these relatively low rates remained stable over time (*P* = 0.20; Figure 3). As immunosuppressive therapy comes with a potential for complications, we examined the secondary diagnoses of complications of medical drug therapy, bundled based on clinical classification algorithms of the NIS. As shown in Figure 3, there was a significant increase in the time period of the study (*P* < 0.001).

## 4. Discussion

The approach to MS has changed with an increasing emphasis on disease modifying therapy and demyelinating lesions as biomarkers of disease. Within the last 20 years, the number of patients receiving such treatments increased from 15% to more than 70% [2, 31]. Despite the consistently positive results of studies on DMT and its increasing use in clinical practice [1, 11, 13–15, 18, 19, 21–23, 25, 31, 32], our findings show increasing hospitalizations. Several potential explanations could contribute to these apparently contradictory trends. First, the increase in hospitalization could be due to a rise in MS prevalence [26]. Only one study has examined time trends in incidence and prevalence of MS in a predefined population and reported stable rates between 1985 and 2000 [33]. More recent studies indicate in increased incidence in US veterans, individuals living in northern Japan, the Netherlands, and Crete [34–37]. Thus, available data are inconclusive but provide at least some support for such an explanation. As the NIS records admissions rather than individual patients, results are likely confounded by multiple admissions of some individuals and do therefore not allow us to draw conclusions about disease prevalence. Second, the threshold for hospital admissions may have changed, potentially accounting for the disproportionate increase in hospitalizations. An analysis of data based on discharge coding cannot directly address this issue. However, reports on annual hospitalization rates for several cohorts of MS patients do showed stable hospitalization rates in defined cohorts within the time period examined, arguing against such an explanation [38–41]. Third, diagnostic criteria for MS were revised during the study time to incorporate advances in imaging techniques [42, 43]. As a result, the need to document more than one distinct attack has become less important and could speed up the diagnosis of the MS and could result in an increase of disease prevalence, driven at least in part by this shift in disease definition. While a potential confounder, this reason seems less likely to explain the ongoing and gradual rather than stepwise rise in admissions observed in our study. Fourth, the spectrum of DMT includes immunomodulation, which could be associated with potential adverse effects, necessitating hospitalization [44, 45]. Consistent with such a potential negative impact of medical interventions, admissions for complications of medical drug therapy increased over time. However, the data do not implicate DMT specifically, as they do not include any information about the actual use of DMT. In addition, therapies for coexisting illnesses or symptom-oriented MS treatments, such as the need for bladder catheterizations in patients with neurogenic bladder, similarly contribute to this trend. When followed over a 12-month period, patients receiving DMT had lower hospitalization rates than those who were not on DMT [39]. Adherence to DMT is associated with lower admission rates [40]. Thus, adverse effects to DMT may account for some hospitalizations but are unlikely to be the primary driver of the observed increase in admissions and are thus also unlikely to explain the rise in admissions due to complications of medical therapy. Lastly, it is certainly possible that other economic or administrative changes in the healthcare system from insurance coverage to reimbursement and requirements for rehabilitative services or institution of home care contributed to our findings [25, 46]. 

We did not only see rising hospitalizations but also stable rates of discharge to ongoing home care or nursing home transfers, which served as surrogate measures of disability. This pattern remained unchanged, when we separately examined different age groups to account for potential skewing of data due to slight changes in the age distribution of hospitalized patients. Similarly, urinary tract infections, which were among the most common secondary diagnoses and correlate with the development of neurogenic bladder [8], did not decrease during the time frame of the study. Finally, the rate of procedures associated with even further progression of functional impairment such as debridement of cutaneous lesions and insertion of gastrostomy tubes was relatively uncommon, but also remained unchanged.

As the majority of MS patients do not require inpatient therapy and as DMT is associated with fewer hospitalizations [39, 40], our approach with a focus on inpatient management skews data and may well underestimate the benefit of DMT. However, descriptions of disability in larger cohorts of outpatients indicate comparable rates of progression to difficulties with ambulation over time. Two decades ago, about one 25–30% of MS patients progressed to the point of needing a cane or wheelchair over 10–15 years [2, 3]. Consistent with this report, one quarter of MS patients studied in the mid 1990s required a wheelchair [47]. Results obtained in 2007 were similar showing that 38% of working age adults with MS at least intermittently relied on wheelchairs [48]. In a cohort of patients undergoing DMT, 20% used a wheelchair in 2006 [49]. The fact that these cohorts were recruited in different areas, were not evaluated and/or treated similarly, limits our ability to draw conclusions. Looking at data from a single center, a similar pattern emerged from a retrospective review of large patient cohorts enrolled between 1975 and 1995 and followed for at least 15 years; the fraction of patients progressing to a more advanced disability stages remained stable at around 25% and did not show a clear drop during time periods that included the introduction and adoption of DMT [50]. While puzzling, prior cost-benefit analyses came to comparable conclusions. Using data obtained in a cohort of patients receiving DMT, Forbes et al. calculated that 18 patients would need to be treated for 30 months in order to delay wheelchair dependence by 9 months [51]. Other modeling approaches also concluded that the relatively modest gains were associated with high healthcare costs [52, 53]. 

We recognize that this study has several important limitations. First, the exclusive focus on inpatient therapy skews data by definition and may well underestimate the benefit of DMT, as already discussed above. Nonetheless, admission rates of cohorts receiving DMT range around 10%, demonstrating an ongoing need for more intense therapy in this group [39, 40]. While about 30% lower than in groups not treated with or adhering to DMT, we still expect a contribution of this subgroup to the overall data. The data used in our investigation as well as in many other epidemiological studies did not include information related to DMT therapy. Therefore, findings cannot be directly related to DMT and interpretations are solely based on parallel time trends. Such parallel trends provide arguments for inferences, which will remain speculative and which do certainly not prove causality. While we focused our discussion on the increasingly widespread adoption of DMT, the time period also witnessed a possible increase in the incidence of MS and changes in diagnostic criteria. Only detailed and prospective cohort studies with extensive data acquisition on treatment, complications, and outcomes can truly address this point. However, the relatively low likelihood of some adverse outcomes (e.g., gastrostomy placement) will make such investigation not only difficult but also prohibitively expensive. We also cannot control for repeated admissions of individual patients, which may bias data. Lastly, we chose a time period that followed the introduction of DMT and thus do not have a true control period without any such therapy. However, the decade between 2001 and 2010 witnessed a widespread adoption of DMT into clinical practice as described above, motivating us to investigate potential parallel developments.

## 5. Conclusion

Our data demonstrate that admission continued to increase disproportionally during a time period that witnessed the widespread adoption of DMT in MS. Similarly, surrogate measures of functional impairment and disease-related complications remained stable during a 10-year period. While the mere temporal coincidence does not allow conclusions about causal relationships, the data may raise questions about benefits of DMT and highlight the ongoing need for symptomatic and supportive interventions.
